# ﻿A new monotypic genus of cobweb spiders from the Russian Far East (Araneae, Theridiidae)

**DOI:** 10.3897/zookeys.1195.118632

**Published:** 2024-03-15

**Authors:** Yuri M. Marusik, Kirill Y. Eskov

**Affiliations:** 1 Institute of Biological Problems of the North, Far Eastern Branch, Russian Academy of Sciences, Portovaya Str., 18, Magadan, 68500, Russia Institute of Biological Problems of the North, Far Eastern Branch, Russian Academy of Sciences Magadan Russia; 2 Altai State University, Lenina Pr., 61, Barnaul, RF-656049, Russia Altai State University Barnaul Russia; 3 Department of Zoology & Entomology, University of the Free State, Bloemfontein, 9300, South Africa University of the Free State Bloemfontein South Africa; 4 Borissiak Paleontological Institute, Russian Academy of Sciences, Profsoyuznaya Str., 123, Moscow, 117647, Russia Borissiak Paleontological Institute, Russian Academy of Sciences Moscow Russia

**Keywords:** *Anelosimus* clade, Aranei, distal theridiids, Kunashir Island, Kuril Islands, new species

## Abstract

A new theridiid spider, *Knoflachiakurilensis***gen. et sp. nov.**, is described from the Kuril Islands (Kunashir). The new genus belongs to the ‘*Anelosimus* clade (clade 24)’ of Agnarsson (2004). A pair of raised, fused setal sockets on the cheliceral promargin adjacent to the fang base was found to be another synapomorphy of all the ‘distal theridiids’ (the ‘elongated central claw clade (clade 33)’: argyrodins, ‘*Anelosimus* clade’ and theridiins). *Knoflachiakurilensis***sp. nov.** demonstrates a male polymorphism similar to some *Anelosimus* Simon, 1891 species (e.g., *A.studiosus* (Hentz, 1850)).

## ﻿Introduction

Theridiidae, or cobweb spiders, is the fourth largest family of the order. Currently, the family includes 2542 recent species placed in 124 genera. Among species-rich families with over 1000 species, it has the highest species/genus ratio 20.5 (WSC 2023). Worldwide Theridiidae genera were considered in three publications: Levi and Levi (1962), Agnarsson (2004) and Vanuytven (2021). Theridiidae of Far East Asia are relatively well studied due to revisions and surveys of Chinese, Japanese and Korean theridiids (Zhu 1998; Yoshida 2009; Kim 2021).

Over 30 years ago, the first author collected in Kunashir Island (Kuril Islands) a large series, over 200 specimens, of brightly-coloured theridiids, which he failed to identify to genus level. Recent attempts involving SEM microscopy allow us to recognize that the species is related to *Anelosimus* Simon, 189, a large genus with 75 named species (WSC 2023). The genus has chiefly a Pantropical distribution with 12 species occurring in Asia, from Indonesia northward to Korea and Japan (Zhang et al. 2011: fig. 19). The specimens from Kunashir have the habitus, colouration, pattern and copulatory organs different from *Anelosimus* species occurring in Asia. Comparison of somatic characters and conformation of copulatory organs between species from Kunashir Island with genotype, *A.eximius* (Keyserling, 1884) known from the Neotropics led us to the conclusion that the new species should be placed in a separate new genus. The goal of this paper is to provide detailed descriptions of the species and genus and to trace the position of the new genus among Theridiidae lineages.

## ﻿Material and methods

SEM images were taken on a Tescan Vega2 and a Tescan Vega3 scanning electron microscope in the Palaeontological Institute (Moscow), operated in high vacuum mode at accelerating voltages of 10–20 kV, using SE and BSE detectors. Specimens were gradually dehydrated in 100% ethanol, dried, and sputter-coated with gold–palladium. Light microscopy photographs were obtained using an Olympus Camedia E‐520 camera attached to an Olympus SZX16 stereomicroscope in the Zoological Museum of the University of Turku. Digital images of different focal planes were stacked with Helicon Focus 8.1.1. All measurements are given in millimeters. The holotype will be deposited in the Zoological Museum of the Moscow State University (**ZMMU**) and paratypes will be shared between the ZMMU and the Zoological Institute (**ZISP**) in St. Petersburg. Abbreviations of leg segments: Fe – femur, Mt – metatarsus, Pa – patella, Ta – tarsus, Ti – tibia.

## ﻿Taxonomy

### ﻿Class Arachnida Cuvier, 1812

**Order Araneae Clerck, 1757**

**Family Theridiidae Sundevall, 1833**

#### ﻿ Knoflachia gen. nov.

Taxon classificationAnimaliaAraneaeTheridiidae

43AEB09C-81D3-53C5-9AB0-4B7CAA3F59F0

https://zoobank.org/B5704C12-015F-4408-A670-CE9CB44CA6CB

##### Etymology.

Named after Barbara Knoflach (Innsbruck, Austria), an outstanding expert in theridiid taxonomy. Gender is feminine.

##### Type species.

*Knoflachiakurilensis* sp. nov.

##### Diagnosis

**(comparison of the generotype).** The new genus is most similar to *Anelosimus* Simon, 1891 in both copulatory organ characters (cymbial mesial margin with an incision; conductor with a groove for a distal portion of the spiral embolus) and somatic characters (male leg I extremely elongated, femur longer than carapace). It differs from the latter by: 1) carapace uniformly orange, abdomen uniformly black (vs carapace pale coloured with dark medial strip, abdomen with characteristic leaf-shaped pattern); 2) carapace cuticle rugose (vs smooth); 3) fovea transversal (vs rounded); 4) pars cephalica mildly sloping (vs not elevated, flat); 5) eye area not projected (vs projected); 6) male prosomal stridulatory ridges (PSR) separated into two patches (vs continuous); 7) female PSR absent (vs weak); 8) sternum equilateral triangle (vs elongated orthogonal); 9) labium fused with sternum (vs separated by distinct seam); 10) bristles of the tarsus IV comb flattened and straight (vs conical and hooked); 11) tarsus IV central claw subequal to laterals in length, thickness and shape (vs elongated, thin and S-shaped); 12) tarsal organ clearly proximal: 0.33–035 (vs slightly distal: 0.55–0.60 to slightly proximal: 0.45); 13) abdominal stridulatory pick row (SPR) setal bases strongly elongated, keeled in male, and dome-like in female (vs moderately elongated and rounded in both sexes); 14) male palpal tibia spoon-like, extremely enlarged, covers more than a half of the bulb (vs cyathiform, usual for theridiids); 15) male palpal tibia with only 2 retrolateral trichobothria (vs 2 retrolateral and 1 prolateral trichobothria); 16) cymbial mesial margin incision fold-like (vs semicircular notch); 17) the tegular apophysis (Ta) is a curved spine (vs not pointed); 18) conductor semimembraneous, its groove forming a sheath for a distal portion of the embolus (vs not membranous, its grove for embolus more shallow); 19) tip of embolus unmodified (vs. modified); 20) epigynal plate smooth (vs ridged); 20) copulatory openings located in two foveae separated by a septum (vs. foveae and septum absent); 21) each fovea with a spiral ridge (epigynal plate with transverse ridges); 22) copulatory ducts (Cd) coiled (vs. not coiled); and 23) receptacles dumbbell-shaped located inside loops of copulatory ducts (vs. oval, not surrounded by copulatory ducts).

##### Description.

**Figure 1.**
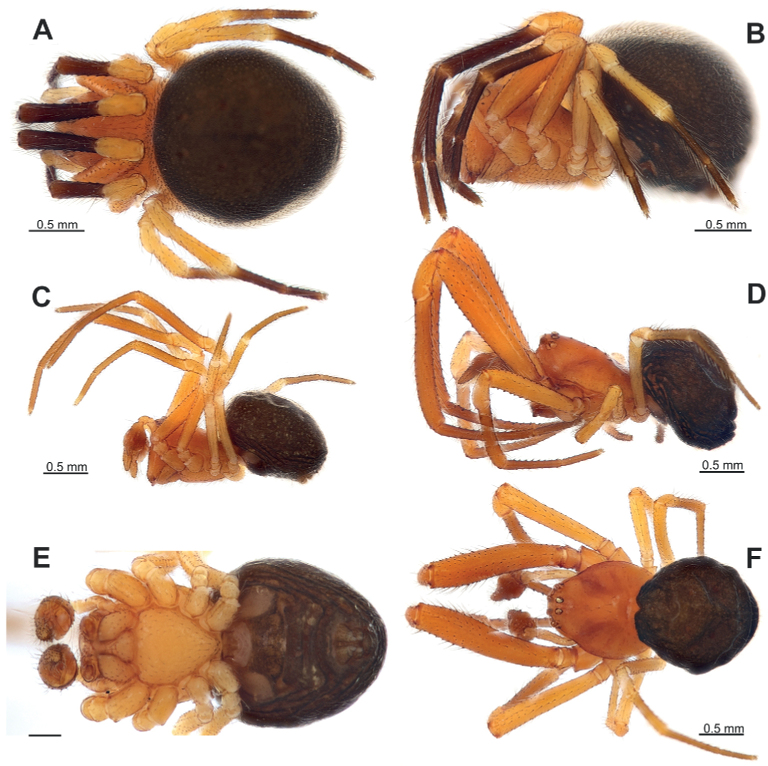
General appearance of *Knoflachiakurilensis* sp. nov. **A, B** female **C, F** male **A, F** dorsal **B–D** lateral **E** ventral **C**, **D** showing the size difference of males. Scale bars: 0.5 mm (**A–D, F**); 0.2 mm (**E**).

**Figure 2.**
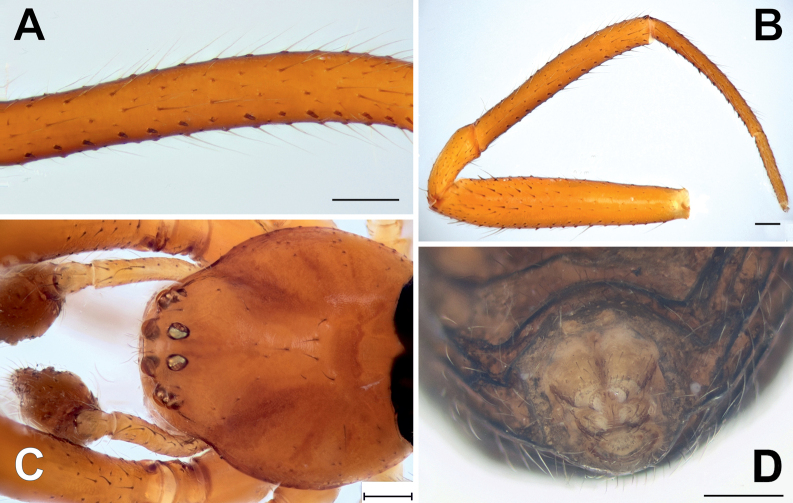
Somatic characters of *Knoflachiakurilensis* sp. nov. **A** part of male tibia I, showing modified ventral seta **B** whole male leg I **C** male prosoma, dorsal **D** posterior part of the female abdomen, ventral, cellular setae indistinct. Scale bars: 0.2 mm.

Small (1.8–2.85) brightly coloured (orange and black) with unmodified carapace and abdomen in both sexes (Fig. 1A–F) and modified leg I in male (Figs 1D, F, 2A, B).

**Figure 3.**
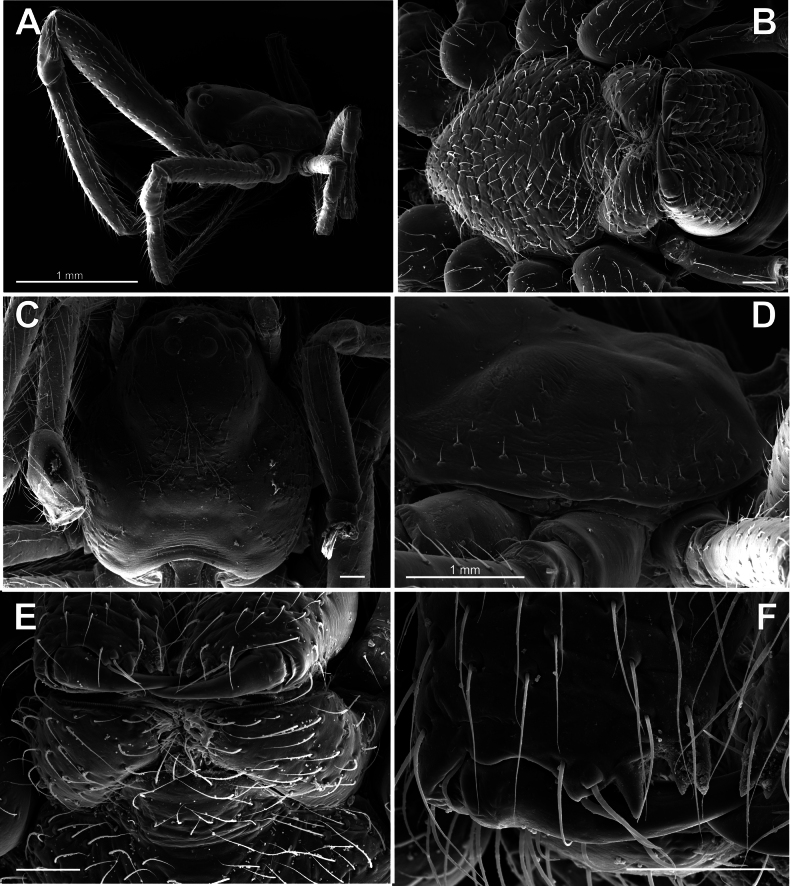
Prosoma of *Knoflachiakurilensis* sp. nov. **A** male carapace and legs, lateral view **B** female sternum **C** female carapace, dorsal view **D** lateral margin of male carapace **E** female labium **F** cheliceral promargin of female (note fused setal socket adjacent to fang base).

**Figure 4.**
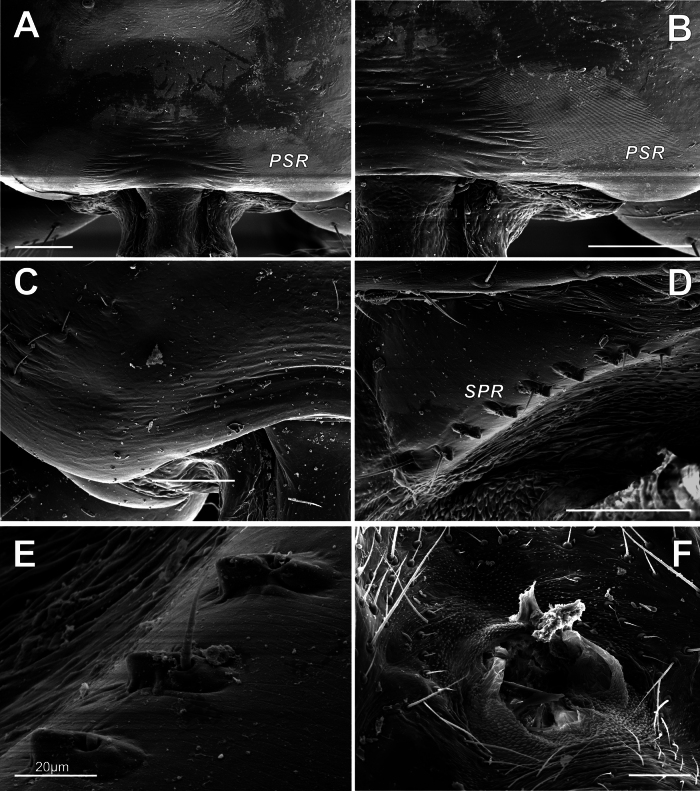
Prosoma-abdomen stridulatory mechanism in *Knoflachiakurilensis* sp. nov. **A** posterior margin of male carapace, prosomal stridulatory ridges **B** the same, enlarged **C** posterior margin of female carapace (note absence of PSR) **D** male abdominal stridulatory pick row **E** the same, enlarged **F** female abdominal stridulatory pick row (note dome-like setal bases of SPR, the same as surrounding setal bases). Abbreviations: PSR – prosomal (carapace) stridulatory ridges; SPR – stridulatory pick row.

***Carapace*** – rounded, almost as wide as long, moderately high, pars cephalica slightly elevated, clypeus vertical; fovea shallow, transversal (Figs 2C, 3A, C, D); 8 medium-sized eyes, AME same as others (ca 1/3 of clypeus height), lateral eyes adjacent, eye area not projected (Figs 2C, 3A, C); carapace pars stridens consist of two separated patches of regular parallel fine ridges in male (Fig. 4, B) indistinct under light microscope (Fig. 2C) and completely absent in female (Fig. 4C,); carapace cuticle rugose, setal bases elevated (Fig. 3C, D).

***Sternum*** – almost equilateral triangle (Figs 1E, 3B); sternal cuticle rugose, setal bases elevated (Fig. 3E).

***Labium*** – sub-rectangular, completely fused to sternum (Fig. 3B, E).

***Chelicera*** – unmodified, without humps; promargin in both sexes with 3 teeth (2 basal fused) and pair of raised, fused setal sockets adjacent to fang base (Fig. 3E, F); cheliceral cuticle rugose, setal bases elevated (Fig. 3F).

**Figure 5.**
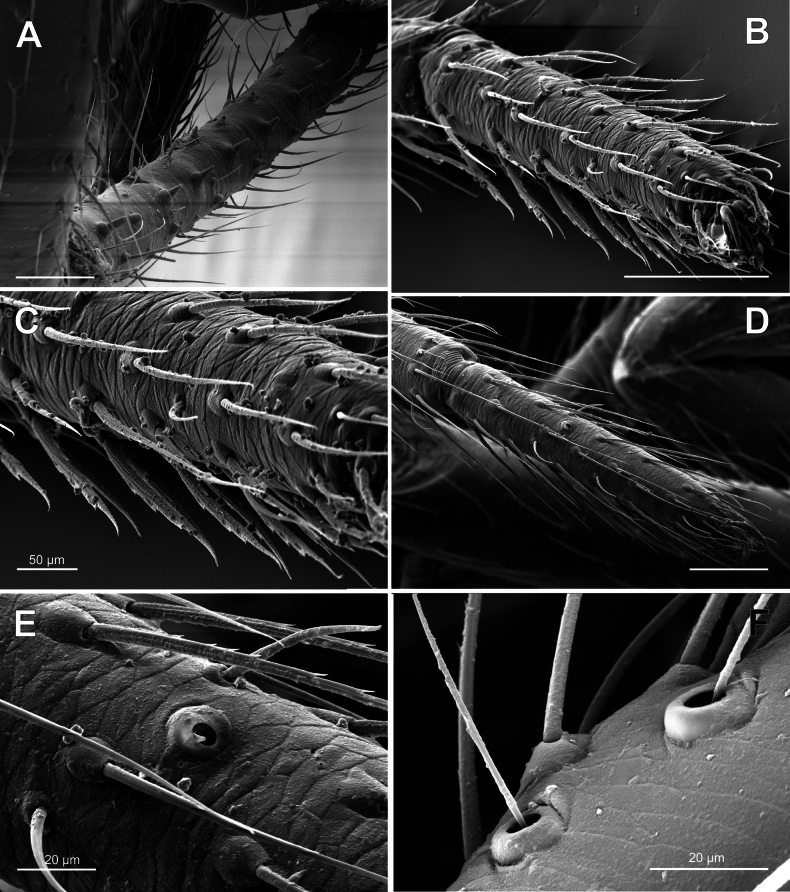
*Knoflachiakurilensis* sp. nov.: leg setae, tarsal organ and trichobothria **A** ventral macrosetae of male metatarsus I **B** tarsus IV comb of female **C** same, enlarged **D** tarsal organ of female leg II **E** same, enlarged **F** trichobothrial bases of male palpal tibia.

**Figure 6.**
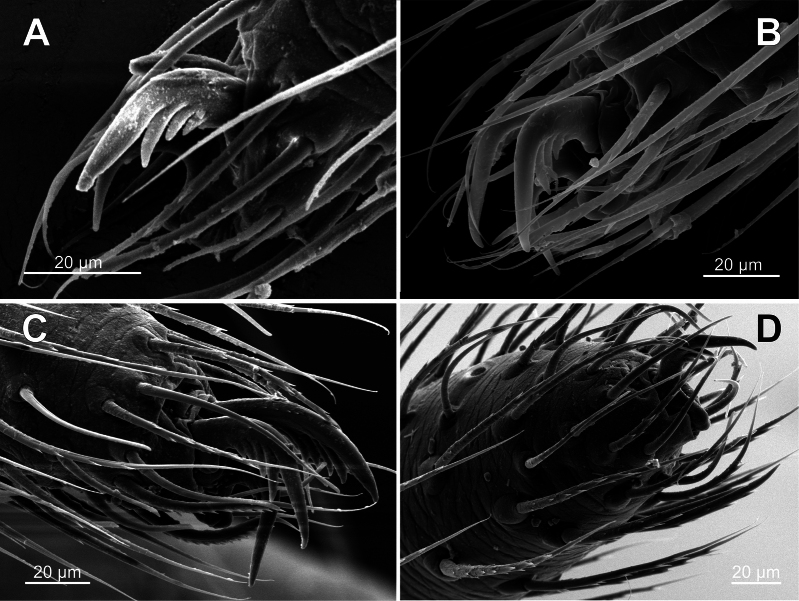
*Knoflachiakurilensis* sp. nov.: tarsal claws **A** tarsus IV of male **B** tarsus IV of female **C** tarsus II of female **D** female palpal claw.

***Legs*** – Leg formula 1243 in males and 1423 in females, leg I of male extremely long and stout, Fe I can be almost 1.5 times longer than carapace length (Figs 1A–D, 2A) in large specimens; distal part of femur 1.5 times wider than proximal; metatarsus and tibia I in large specimens with 2 ventral rows of tubercles, surmounted by robust blunt suberect macrosetae (Figs 2A, B, 5A); bristles of tarsus IV theridiid comb flattened and straight, not hook-like (Fig. 5B, C); tarsus IV central claw subequal to laterals by length, thickness and shape in both sexes, and not distinguished from claws of other leg pairs (Fig. 6A–C); metatarsal trichobothria 1-1-1-0, bothria dome-like (Fig. 5F), usual for theridiids (Eskov and Marusik in prep.); tarsal organ clearly proximal (0.33–0.35), its opening large, more than setal sockets (Fig. 5D, E); leg cuticle imbricate (Fig. 5E).

***Female palp*** – full-segmented; palpal tibia with 2 trichobothria; palpal claw simple, non-semipalmate, strongly dentated (Fig. 6D).

**Figure 7.**
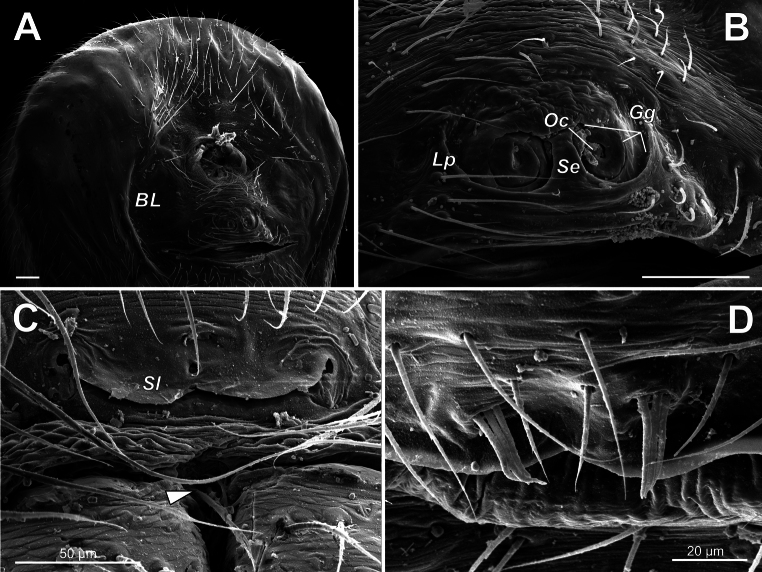
Abdomen of *Knoflachiakurilensis* sp. nov.: female (**A–C**) and male (**D**) **A** epigastric region of female **B** epigyne **C** colular setae (arrow) and tracheal spiracle **D** socketed epiandrous spigots. Scale bars: 0.1 mm if not otherwise indicated. Abbreviations: *BL* – booklung covers; *Gg* – guiding groove; *Lp* – latero-posterior rim of epigyne; *Oc* – copulatory opening; *Se* – septum; *Sl* – lobe of spiracle opening.

**Figure 8.**
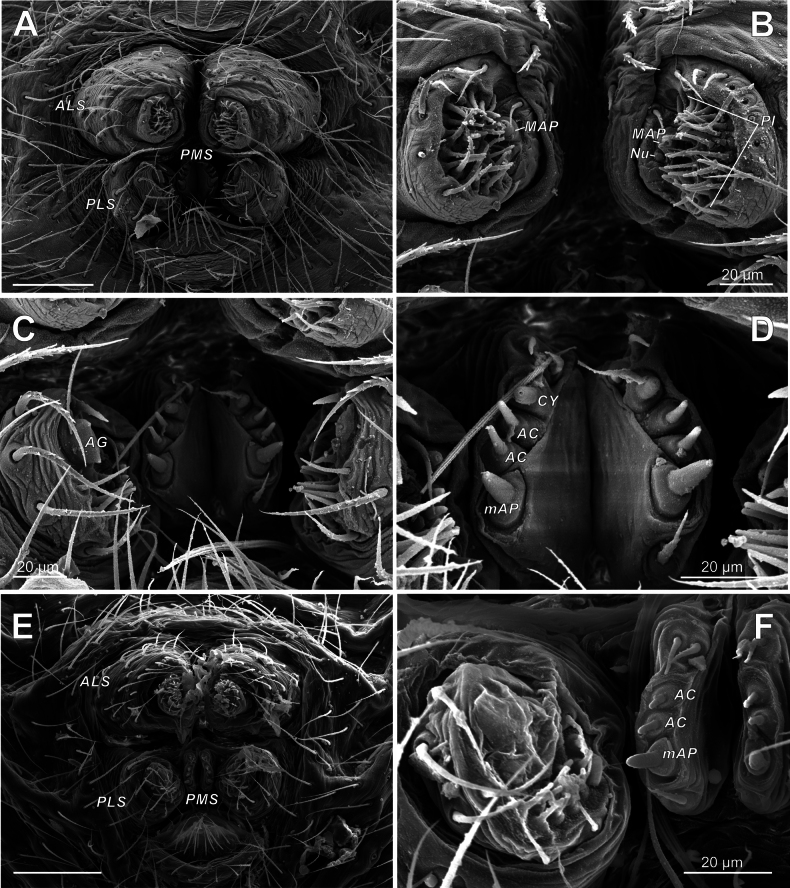
Spinnerets of *Knoflachiakurilensis* sp. nov. **A** female spinnerets **B** female ALS **C** female PLS and PMS **D** female PMS **E** male spinnerets **F** male PLS and PMS (note absence of CY). Abbreviations: *AC* – aciniform gland spigot(s); *AG* – aggregate gland spigot(s); *ALS* – anterior lateral spinneret; *CY* – cylindrical gland spigot(s); mAP – minor ampullate gland spigot(s); *MAP* – major ampullate gland spigot(s); *Nu* – nubbin; *PI* – piriform gland spigot(s); *PLS* – posterior lateral spinneret; *PMS* – posterior median spinneret.

***Abdomen*** – more or less globular; pedicel area with suprapedicillate dorsal (11 o’clock) proprioceptor setae (Figs 4F, 7A); stridulatory pick row (SPR) lateral of pedicel, regular, not curved, consist of few (< 7) setae (Fig. 4D, F); SPR setal bases strongly elongated and keeled, tetragonal in profile, with setae vertically protruded from its middle portion in male (Fig. 4D, E), and rounded, dome-like in female (Fig. 4F); epiandrous gland spigots arranged in a pair of distinct depressions (sockets), 3 spigots per socket (Fig. 5D); wide tracheal spiracle near spinnerets, not straight, with pair of lobes (Sl, Fig. 7C); colulus absent, but median pair of colular setae persists (Fig. 7C); ALS piriform field small, less than 20 spigots (Fig. 8A, B, E); PLS posterior AG spigot enlarged and flattened, subquadrate in profile (Fig. 8A, C); PMS with 3 spigots (1 mAP + 2 AC) in male (Fig. 8E, F) and 4 spigots (1 mAP + 2 AC + 1 CY) in female (Fig. 8A, E); abdominal cuticle fingerprint (Fig. 7A), booklung cuticle smooth.

**Figure 9.**
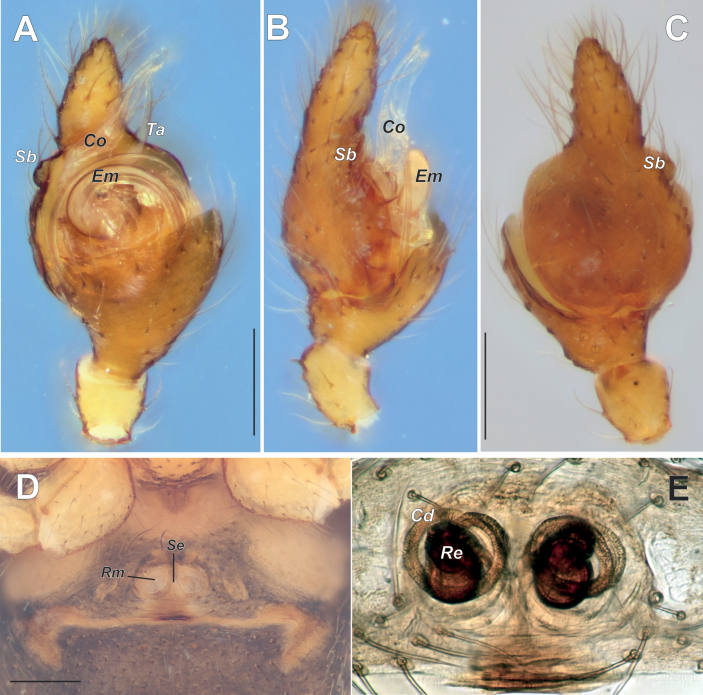
Male palp (**A–G**) and epigyne (**D–E**) of *Knoflachiakurilensis* sp. nov. **A–C** ventral, prolateral and dorsal **D** intact, ventral **E** macerated, dorsal. Scale bars: 0.2 mm. Abbreviations: *Cd* – copulatory duct; *Co* – conductor; *Em* – embolus; *Re* – receptacle; *Rm* – round part of epigyne; *Sb* – cymbial small bulge; *Se* – septum; *Ta* – tegular apophysis.

**Figure 10.**
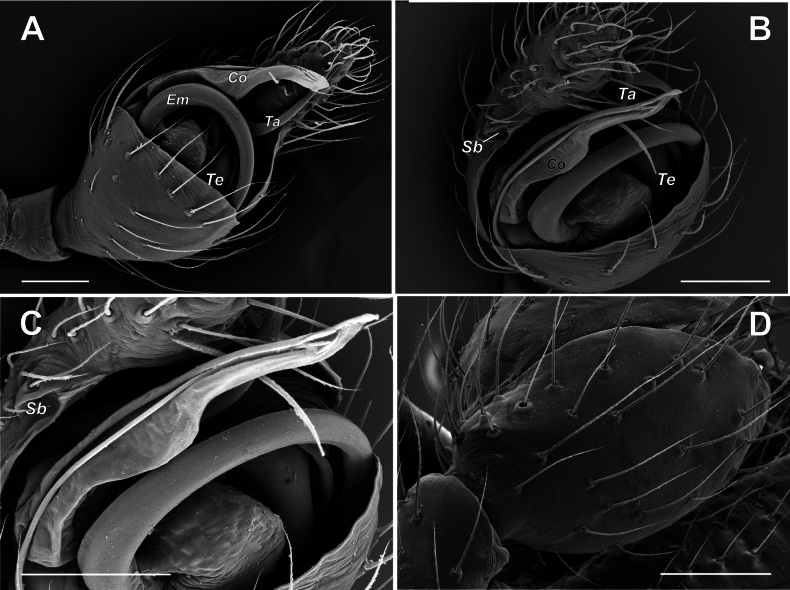
Male palp of *Knoflachiakurilensis* sp. nov. **A** left palp, ventral view **B** left palp, ventro-mesial view **C** the same, enlarged (note grooved conductor, forming a sheath for a distal portion of embolus, and fold-like excavation on cymbial mesial margin) **D** tibia of right palp, dorso-ectal view. Scale bars: 0.1 mm. Abbreviations: *Co* – conductor; *Em* – embolus; *Sb* – cymbial small bulge; *Ta* – tegular apophysis; *Te* – tegulum.

**Figure 11.**
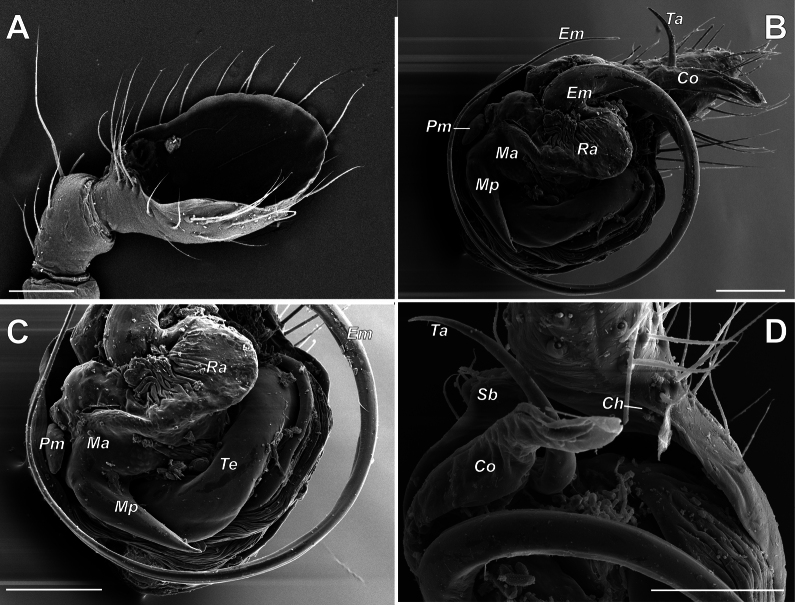
Left male palp with removed tibia of *Knoflachiakurilensis* sp. nov. **A** separated tibia, inner surface **B** bulb and cymbium, ventral **C** same, proximal portion **D** same, distal portion (note ectal cymbial hood and mesial cymbial excavation). Scale bar: 0.1 mm if not otherwise indicated. Abbreviations: *Ch* – cymbial hood; *Co* – conductor; *Em* – embolus; *Ma* – median apophysis; *Mp* – posterior part of *Ma*; *Pm* – prolateral part of *Ma*; *Ra* – radix; *Sb* – cymbial small bulge; *Ta* – tegular apophysis; *Te* – tegulum.

***Male palp*** – patella short, almost as wide as long, 2.4 times shorter than tibia; tibia spoon-like, extremely enlarged, ca 2/3 of cymbial length, covers more than half of proximal part of bulb (Figs 9A, 10A), with 2 retrolateral trichobothria (Fig. 10D) and with several setae on its inner surface (probably artifact) (Fig. 11A) and; cymbium 1.9 times longer than wide, with round proximal part and finger like tip slightly bent retrolaterally; cymbial mesial margin distally with small bulge (Sb) (when observed with light microscope: Fig. 9A–C), which is dissected longitudinally by fold-like excavation (when observed with SEM: Figs 10B, C, 11D); cymbial ectal margin distally with groove-like cymbial hood (Ch) (Fig. 11D). Bulb as long as wide, with relatively small tegulum (Te), tegular apophysis (Ta) appears as simple curved, weakly sclerotized spine (Figs 9A, 10A, B, 11B, D); elongated hyaline conductor (Co) with groove serving as sheath for distal portion embolus (Fig. 10B, C); embolus filamentous coiled forming loop ca. 400°, (Fig. 11B); radix (Ra) and median apophysis (Ma) (we are not sure about homology) in unexpanded palp completely hidden by enlarged retroventral part of tibia (Figs 10A, 11C); median apophysis (?) with 2 arms: large spine like posterior (Mp) and small prolateral (Pm).

***Epigyne*** – as seen by light microscope (Fig. 9D, E): epigynal plate about as long as wide, weakly sclerotized, with pair of round membranous parts (Rm) separated by thin septum; endogyne with long coiled weakly sclerotized copulatory ducts (Cd) and dumbbell-shaped receptacles (Re) standing perpendicular to epigynal plate behind Rm. As seen by SEM (Fig. 7A, B): epigyne with kind of foveae, well delimited by latero-posterior rim (Lp) and septum (Sp). Rim and septum forming guide groove (Gg) for embolus, anteriorly from septum Gg shallower, Gg forms 2 coils (about 720°) and terminates in copulatory opening (Oc).

##### Composition and distribution.

**Figure 12.**
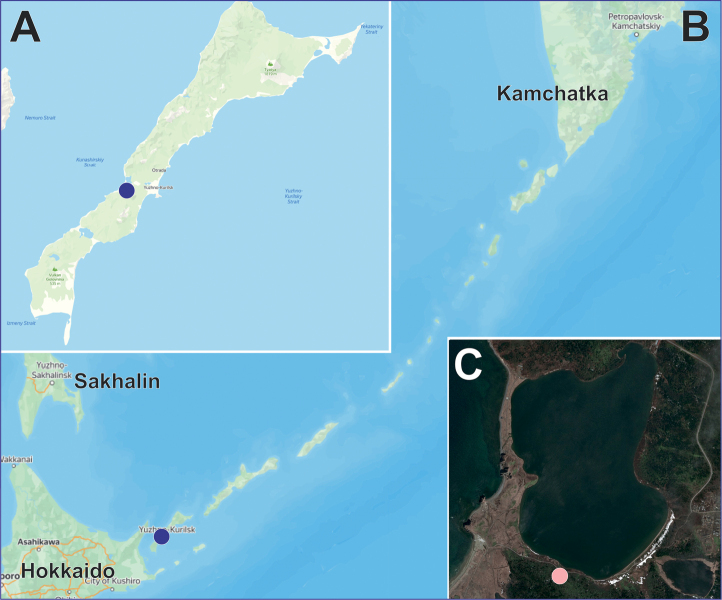
Collecting locality of *Knoflachiakurilensis* sp. nov. **A** Kunashir Island **B** Kurile islands **C** collecting locality.

Only the type species, *K.kurilensis* sp. nov., known only from the type locality (South Kurile islands, Kunashir Island: Fig. 12). The future records of this genus are surely anticipated in other Far East regions such as Hokkaido.

#### ﻿ Knoflachia kurilensis sp. nov.

Taxon classificationAnimaliaAraneaeTheridiidae

53F597C7-39F2-5571-869D-8924F5EF85BF

https://zoobank.org/0272E07A-7614-436B-8FFA-EFFDFF521EB4

Figs 1 , 2 , 3 , 4 , 5 , 6 , 7 , 8 , 9 , 10 , 11 , 12

##### Etymology.

Named after the type locality, Kuril Islands.

##### Material.

***Holotype*** ♂ and ***allotype*** ♀ (ZMMU) and ***paratypes*** 144♂ 1 (♂) 77♀ (ZMMU & ZISP) with label “[KU-123] KUNASHIR Isl., CW part, S shore of Lagunnoye Lake, 44°03′05″N, 145°45′E, sweeping along road, mostly alder bushes, 24.09.1997 Yu. M. Marusik”.

##### Diagnosis.

Same as for the genus. Well differs from other Theridiidae occurring in East Asia by colouration (Fig. 1), epigyne with septum (Fig. 9D) and male palp with strongly enlarged tibia covering half of the bulb (Fig. 9A–C).

##### Description.

Male (holotype). Total length 2.4. Carapace 1.38 long, 1.08 wide; abdomen 1.45 long, 1.35 wide. Prosoma, including legs orange-reddish, legs I as dark as carapace, sternum, mouthparts, and legs II–IV lighter. Abdomen uniformly dark dorsally, venter with lighter booklung covers and area near petiolus. Palp and leg lengths as per Table 1.

**Table 1.** Palp and leg lengths of the male holotype.

♂	**Fe**	**Pa**	**Ti**	**Mt**	**Ta**	**Total**
Palp	0.5	0.17	0.3	–	0.43	1.4
I	1.75	0.65	1.38	1.13	0.6	5.51
II	1.25	0.55	0.85	0.88	0.48	4.01
III	0.8	0.33	0.48	0.58	0.4	2.59
IV	0.95	0.38	0.55	0.75	0.48	3.11

***Palp*** – see genus description.

***Small male*.** Total length 2.13. Carapace 1.0 long, leg I 4.26 (1.38. 0.5, 1.0, 0.88, 0.5). Pattern as in holotype.

***Variations*.** Total length varies from 1.9 to 2.5. At least one male has leg colouration like in females.

**Female** (allotype). Total length 2.75. Carapace 1.08 long, 1.0 wide. Carapace, sternum, mouth parts as in male. Legs I–II with dark tibiae, metatarsi and tarsi, legs III–IV with dark metatarsi and tarsi. Palps with dark tibiae and tarsi. Palp and leg lengths as per Table 2.

**Table 2.** Palp and leg lengths for female allotype.

♀	Fe	Pa	Ti	Mt	Ta	Total
Palp	0.39	0.16	0.21	–	0.34	1.1
I	1.24	0.41	0.87	0.8	0.46	3.78
II	0.99	0.41	0.57	0.57	0.39	2.93
III	0.66	0.27	0.39	0.43	0.34	2.09
IV	1.0	0.37	0.6	0.64	0.4	3.01

***Variations*** – Total length varies from 2.25 to 2.85, colour of abdomen in alcohol from almost black to grey.

##### Natural history.

All specimens were collected in one day by sweeping bushes. The great number of specimens collected in a few hours most likely indicates that they may form colonies, like *Anelosimus*. Some specimens look like penultimate. The presence of only one subadult specimen indicates that the species is monovoltine.

##### Distribution.

Known from the single locality in Kunashir Island (Fig. 12).

##### Male polymorphism.

Males vary in size and relative length of the first leg (cf. Fig. 1C and 1D). Larger males have a relatively longer femur I (femur I/carapace length ratio ca. 1.45 in large male and 1.33 in small). Larger males have a distinct tibial and metatarsal macrosetae on legs I and II (setae standing on tegumental stump-like outgrowth Figs 2A, B, 5A). Size polymorphism is known in several groups of Theridiidae (e.g., *Parasteatodatepidariorum* (C.L. Koch, 1841), *Steatodatriangulosa* (Walckenaer, 1802) [Nentwig et al. 2023]). Large-size polymorphism was documented in *Enoplognathamonstrabilis* Marusik & Logunov, 2002 (see Marusik and Logunov 2002), a species with similarly modified setae on leg I and occurring in Siberia. The carapace of the largest male is 1.7 times longer than in the smallest one. The same type of modified setae is documented in the New World *Anelosimusstudiosus* (Hentz, 1850) (see Agnarsson 2004) and in the East Mediterranean *Enoplognathaparathoracica* Levy & Amitai, 1981 and *E.quadripunctata* Simon, 1884 (see Huseynov and Marusik 2008).

## ﻿Discussion

The following characters of *Knoflachia* gen. nov. should be discussed in more detail, in comparison with other theridiid genera. In the naming (and the numbering) of the theridiid clades we are following Agnarsson’s (2004: fig. 105) cladogram.

1. **Prosoma-abdomen stridulatory mechanism.** “Typically, pairs of elevated setal bases, here called stridulatory picks (SP; the term *plectrum* refers to such stridulatory parts in general) bordering [as a row, SPR] the pedicel on the abdomen interact with ridges on the posterior margin of the carapace (pars stridens)” (Agnarsson 2004: char. 128). This mechanism is characteristic of theridiid spiders, and the presence of SPR was stated as a synapomorphy of the theridiids minus hadrotarsines (‘SPR clade, clade 50’). “Although commonly present in both sexes, both the picks [...] and the ridges [...], are usually much reduced in the female, and the stridulatory role in male courtship shown for a number of species [...] can thus be presumed to be universal” (Agnarsson 2004: p. 474). The sexual dimorphism of this character in *Knoflachia* gen. nov. is extremely strong: SPR setal bases sharply differ in male (longitudinal and strongly keeled: Fig. 4D, E) and in female (dome-shaped, as well as other setal bases: Fig. 4F), while a ridged prosomal pars stridens is present in the male (Fig. 4A, B) and completely absent in the female (Fig. 4C). As regards the male prosomal stridulatory ridges (PSR), the two characters with two modalities may be distinguished here: (1) pars stridens separated into two patches, or continuous (Agnarsson 2004, char. 129); (2) ridges clear and deep, or fine, sometimes irregular (Agnarsson 2004, char. 128). Accordingly, every theridiid genus may be nested by its pars stridens parameters into the four-cell matrix: (A) clear-ridged / continuous; (B) clear-ridged / two-patches-separate; (C) fine-ridged / continuous; (D) fine-ridged / two-patches-separate, as follows: (A) clear-ridged / continuous: *Crustulina* Menge, 1868, *Helvibis* Keyserling, 1884, *Pholcomma* Thorell, 1869, *Robertus* Pickard-Cambridge, 1879, *Tidarren* Chamberlin & Ivie, 1934 (see Agnarsson 2004: figs 42G, 49F, 60F, 66G, 87B, respectively). (B) clear-ridged / two-patches-separate: *Argyrodes* Simon, 1864, *Coleosoma* O. Pickard-Cambridge, 1882, *Enoplognatha* Pavesi, 1880, *Neospintharus* Exline, 1950, *Rhomphaea* L. Koch, 1872, *Steatoda* Sundevall, 1833 (see Agnarsson 2004: figs 32A, 41F. 44H, 57A, 64E, 71F, respectively). (C) fine-ridged / continuous: *Anelosimus* Simon, 1891, *Chrysso* O. Pickard-Cambridge, 1882, *Carniella* Thaler & Steinberger, 1988, *Episinus* Walckenaer, 1809, *Nesticodes* Archer, 1950, *Selkirkiella* Berland, 1924, *Theridion* Walckenaer, 1805 (see Agnarsson 2004: figs 21D, 40F, 36G, 47B, 58D, 67G, 75D, respectively). (D) fine-ridged / two-patches-separate: *Knoflachia* gen. nov. (Fig. 4A, B) only, as it is known. Besides, tetragonal in profile SPR setal bases vertically protruded from its medial portion setae seem to be unique in *Knoflachia* gen. nov. (Fig. 4D, E) (i.e., neither ectally nor mesially directed: Agnarsson 2004, chars 153, 156, 157).
2. **Size of tarsal organ**: “The typical araneoid tarsal organ (all legs and palpi) is about the size of a macrosetal or trichobothrial socket, with the opening clearly smaller than the width of adjacent setae […] Some theridiids (clade 46) have enlarged tarsal organs in which the circumference is equal to or larger than adjacent setal sockets, and the opening is as large or larger than those of setal or trichobothrial sockets” (Agnarsson 2004: char. 198). The tarsal organ opening of *Knoflachia* gen. nov. is clearly larger than those of the setal sockets (Fig. 5E), and it belongs to the ‘enlarged TO clade (clade 46)’, uniting all the non-hadrotarsin and non-latrodectin theridiids.
3. **Tarsus IV central claw vs. laterals.** “The middle tarsal claw of all argyrodines is notably long in both sexes [...]. In most male theridiids the central claw is relatively longer than in females and here the central claw longer than laterals is a synapomorphy for clade 33” (Agnarsson 2004: char. 199). This character (i.e., notably elongated and S-shaped middle claw of the tarsus IV), provided the name to the ‘elongated central claw clade (clade 33)’, united all the ‘distal theridiids’: argyrodins (e.g., *Argyrodes*), ‘Anelosimus clade’ (e.g., *Anelosimus*) and Theridiinae (e.g., *Theridion*) (see Agnarsson 2004: figs 32H, 21C and 76C, respectively). Surprisingly, the middle tarsal claws in *Knoflachia* gen. nov. are unmodified: subequal to laterals in both sexes and on all leg pairs (Fig. 6A–C). However, it seems rather a reversal, unique for the ‘distal theridiids’ (the clade, supported, e.g., synapomorphy (4) – see below).
4. **Setal sockets on cheliceral promargin.** A pair of raised, fused setal sockets on the cheliceral promargin adjacent to the fang base (Fig. 3E, F) seems an important character, overlooked (or at least underestimated) by earlier workers. Such fused sockets are present in all the ‘distal theridiids’: Argyrodinae (e.g., *Argyrodes*), ‘ *Anelosimus* clade’ (e.g., *Anelosimus*) and Theridiinae (e.g., *Theridion*) (see Agnarsson 2004: figs 33G, 22E and 80C, respectively), and absent in all the ‘basal theridiids’: Hadrotarsinae (*Dipoena* Thorell, 1869 and *Euryopis* Menge, 1868), Latrodectinae (*Latrodectus* Walckenaer, 1805 and *Steatoda*) and Spintharinae (*Spintharus* Hentz, 1850 and *Thwaitesia* O. Pickard-Cambridge, 1881) (see Agnarsson 2004: figs 5B, 8E, 55E, 72E, 70E and 84D, respectively). As regards Pholcommatinae, with its intermediate position in Agnarsson’s (2004: fig. 105) cladogram, it incorporates the genera with fused setal sockets (e.g., *Cerocida* Simon, 1894), as well as with unfused ones (e.g., *Enoplognatha*) (see Agnarsson 2004: figs 37D and 45B, respectively). So, the fused setal sockets on the promargin (as in *Knoflachia* gen. nov.) are an unambiguous synapomorphy of the ‘distal theridiids’, and autapomorphies of several pholcommatin genera. On the one hand, it is important support of the ‘elongated central claw clade (clade 33)’ by Agnarsson (2004: 462). On the other hand, it may be an additional argument on the heterogenity of Pholcommatinae (“The composition of this subfamily is uncertain”: Agnarsson 2004: p. 468). For instance, the well-supported clade uniting *Carniella*, *Theonoe*, *Robertus* and *Pholcomma* (Knoflach 1996; Agnarsson 2004: 463) is sharply distinguished by a trichobothrial base morphology not only from all the rest of the pholcommatins, but from all the theridiids (Eskov and Marusik in prep.).
5. **Colulus and colular setae.** “The colulus is generally considered homologous to the cribellum [...]. It has been lost frequently in araneoids. Here the loss of a colulus is synapomorphic for *Anelosimus* plus theridiines (clade 25, termed the lost colulus clade)” (Agnarsson 2004: char. 172), and later: “The colulus usually bears some setae, including a distinctive median pair of long setae [...]. Even when the colulus is absent this pair of setae often persists [...]. These setae are lost distally in the lost colulus clade, a theridiine synapomorphy” (Agnarsson 2004: char. 174). In *Knoflachia* gen. nov. the colulus is lost, but a median pair of colular setae persists (Fig. 7C), as well as in, for example, *Anelosimus* (see Agnarsson 2004: fig. 16E). So, it belongs to the ‘lost colulus clade (clade 25)’ of the ‘distal theridiids’, but not to its terminal branch, the ‘lost colular setae clade (clade 15)’, that is, Theridiinae. Besides the absence of any trace of a colulus, this clade is unambiguously defined by a reduction of the trichobothria number on the male palpal tibia to one and the epiandrous gland spigots spread over the epiandral plate (Agnarsson 2004: p. 470), and *Knoflachia* gen. nov. also lacks all of the listed synapomorphies (see below).
6. **Male palpal tibia trichobothria.** “The number and distribution of trichobothria on the male palpi seem quite informative phylogenetically […]. *Synotaxus* and theridioids primitively have two retrolateral and one prolateral trichobothria […] Reduction to a single retrolateral trichobothria […] (or uniquely in *Carniella* […] to none) occurred at least five times in theridiids, but although homoplasious […], in most instances the reduction is informative […]. The same applies to reduction in prolateral trichobothria” (Agnarsson 2004: char. 18). In the ‘distal theridiids’ the abovementioned plesiomorphous condition ‘2 rl + 1 pl’ is conserved in the Argyrodinae (clade 32) and in the ‘ *Anelosimus* clade (clade 24)’ (see Agnarsson 2004: figs 31G, 24C, respectively). A single retrolateral trichobothria in Theridiinae (clade 15) (e.g., *Theridula* Emerton, 1882, *Thymoites* Keyserling, 1884 and *Tidarren* Chamberlin & Ivie, 1934 – see Agnarsson 2004: figs 81A, 85B, 86B, respectively) is an unambiguous synapomorphy of this clade (Agnarsson 2004: 470). *Knoflachia* gen. nov. possesses two retrolateral and no prolateral trichobothria (Fig. 10D), and this combination seems unique among the ‘distal theridiids’. On the one hand, it is plesiomorphous relative to theridiins (clade 15), with their single retrolateral trichobothria. On the other hand, it is relatively apomorphous ‘ *Anelosimus* clade (clade 24)’, where a prolateral trichobothria persists.
7. **Epiandrous spigots.** “In many orbicularians the epiandrous gland spigots are arranged in irregular transverse rows along the male genital plate [...]. In, others [...], and many basal theridiids, however, the spigots are tightly arranged in two patches, usually in clear depressed sockets [...]. Here, [....] socketed epiandrous spigots are interpreted as synapomorphic for theridiids, with three reversals. A reversal to spigots in rows defines latrodectines [...]; it is autapomorphic in *Ariamnes* [...] and is a synapomorphy for Theridiinae” (Agnarsson 2004: char. 169). As regards the epiandrous spigot pair number, Agnarsson (2004: char. 170) recognized the trend of their reduction, from the plesiomorphic condition (>10 spigots) to the apomorphic condition (<8 spigots). The final point of this trend is the reduction of the spigot number to 2–3. It is quite usual among the ‘spigots in rows’ theridiids: *Latrodectus*, *Ariamnes*, *Thymoites*, *Chrysso*, some *Ameridion* Wunderlich, 1995 and *Thwaitesia* (see Agnarsson 2004: figs 55F, 34G, 85E, 40A, Figs 14F, 83F, respectively), but very rare among ‘socketed spigots’ theridiids: *Faiditus*, some *Anelosimus* (see Agnarsson 2004: figs 48G, 28G) and *Glebych* Eskov & Marusik, 2021 (see Eskov and Marusik 2021: fig. 4F, G). So, the ‘socketed spigots’ of *Knoflachia* gen. nov. (Fig. 7D) is a plesiomorphic condition for the ‘distal theridiids’. On the other hand, the reduction of the spigot number up to 3 is an autapomorphy of this genus.
8. **Labium-sternum connection.** “A distinct seam is sometimes present between the sternum and the labium. Although much used in keys and taxonomy [...], the presence or absence of a seam is extremely homoplasious within theridiids [...] and thus of limited usefulness at higher systematic levels. Here a fused connection is, for example, an ambiguous synapomorphy of Theridiinae [...] but is certainly not characteristic of the clade” (Agnarsson 2004: char. 135). However, the fused labium-sternum connection in *Knoflachia* gen. nov. (Figs 1E, 3B, E) is an informative autapomorphy, distinguishing it from such probable relatives as *Anelosimus* and *Selkirkiella* (Agnarsson 2004: figs 27C, 68A, respectively).
9. **Leg I of male.** The extremely elongated male leg I in *Anelosimus* (see Vanuytven 2021: figs B.16, B.18) is provided with a unique thickened ventral macrosetae on metatarsus I (Agnarsson 2004: char. 188, fig. 26F). The same features found in *Knoflachia* gen. nov. (Figs 2A, B, 5A) may be interpreted as a synapomorphy of these genera in limits of the ‘ *Anelosimus* clade (clade 24)’.
10. **Cymbial mesial margin.** “Many *Anelosimus* have a distinct incision on the cymbial mesial margin [...] and on this cladogram the feature is a synapomorphy of clade 22 [*Anelosimus* exclusive of *A.vittatus* and *A.pulchellus*]” (Agnarsson 2004: char. 25). The cymbial mesial margin of *Knoflachia* gen. nov. is modified too, but its incision is fold-like (Figs 10B, C, 11D), unlike the *Anelosimus* notch (see Agnarsson 2004: figs 17D, 20A).
11. **Tarsus IV ventral setal comb.** “Tarsal comb bristle morphology varies greatly […] Various theridiids have simple straight bristles […], while in others the serrations form curved hooks” (Agnarsson 2004: char. 195). The straight and flattened, with clear ‘theridiid grooves’, comb bristles of *Knoflachia* gen. nov. (Fig. 5B, C) clearly differ from the hooked and conical bristles of *Anelosimus* species (see Agnarsson 2004: figs 19E, 23E) and are similar rather to those of *Parasteatodawau* (Levi, Lubin & Robinson, 1982) (see Agnarsson 2004: fig. 12F, as *Achaearaneaw.*).
12. **Cuticle.** “Theridiid taxonomic work often refers to carapace (and/or sternum) ‘rugosity’. However, ‘rugosity’ differs between taxa […]. Although autapomorphic here, these conditions will probably be generic synapomorphies. *Steatoda* and *Crustulina*, synapomorphically, have rugosity caused by the elevation of setal bases […]. Most theridiid carapaces are relatively smooth” (Agnarsson 2004: char. 123); “As on the carapace, the setal bases of the sternum of *Steatoda* and *Crustulina* are elevated” (Agnarsson 2004: char. 139). *Knoflachia* gen. nov. possess, undoubtedly convergently, the carapace/sternum ‘rugosity’, caused by the elevation of setal bases (Fig. 3B–E), of the same type as mentioned above for *Steatoda* and *Crustulina* (see Agnarsson 2004: fig. 71D–F), whereas all members of the ‘ *Anelosimus* clade (clade 24)’ possess a smooth cuticle (see Agnarsson 2004: figs 19F, 21B).
13. **Tarsal organ position.** A few theridiids clearly have a proximal tarsal organ, for example, 0.3 in *Glebych* (Eskov & Marusik, 2021: fig. 4D). The majority of theridiids have clearly distal TO (> 0.6); in *Anelosimus* TO position varying from ‘slightly distal (0.55–0.60) on tarsi I and II’ to “slightly proximal (0.45) on IV’ (Agnarsson et al. 2015: 39).

So, clearly a proximal TO in *Knoflachia* gen. nov. (0.33–035) (Fig. 5D) is a remarkable character of this genus.

## ﻿Conclusions

The position of *Knoflachia* gen. nov. in Agnarsson’s (2004: fig. 105) cladogram of the family can be clarified as follows:

The presence of a prosoma-abdomen stridulatory mechanism (1) and a large opening of the tarsal organ (2) places *Knoflachia* gen. nov. in the ‘SPR clade (clade 50)’ and the ‘enlarged TO clade (clade 46)’, respectively, uniting all non-hadrotarsin and non-latrodectin theridiids.

Despite the unmodified tarsal claws (3), *Knoflachia* gen. nov. should be nested in the ‘elongated central claw clade (clade 33)’, uniting all ‘distal theridiids’, due to the presence of a pair of fused setal sockets on the cheliceral promargin (4), a newly found synapomorphy of this clade.

The position of *Knoflachia* gen. nov. within the ‘distal theridiids’ is determined by its colular characters: colulus is lost, but a median pair of colular setae persists (5). So, it belongs to the ‘lost colulus clade (clade 25)’, uniting all the non-argyrodin ‘distal theridiids’, but does not belong to its terminal branch, the ‘lost colular setae clade (clade 15)’, i.e., Theridiinae.

The non-inclusion of *Knoflachia* gen. nov. to the ‘lost colular setae clade (clade 15)’ is supported by the lack of all theridiin synapomorphies: its colular setae persists (5), the number of male palpal tibia trichobothria is not reduced to a single one (6), and its epiandrous spigots are still socketed, not spread over the epiandral plate (7).

In summary, *Knoflachia* gen. nov. belongs neither to Argyrodine (i.e., the basal clade of ‘distal theridiids’), nor to Theridiinae (i.e., the terminal clade of ‘distal theridiids’). It should nest within the remaining ‘distal theridiid’ group, that is, ‘*Anelosimus* clade (clade 24)’, which was not attributed by Agnarsson (2004: p. 470) to conventional subfamilies due to its probable paraphyly.

Within the ‘*Anelosimus* clade’, *Knoflachia* gen. nov. possess several autapomorphies: reduction of the male palpal trichobothria up to 2 retrolateral, vs. 2 retrolateral and 1 prolateral (6), reduction of the epiandrous spigots to 3 per socket (7), and the labium fused to the sternum (8). It seems most close to the genus *Anelosimus* by the male leg I modification (9) and the cymbial mesial margin incision (10); however, it is distinguished by numerous characters, for example, prosomal stridulatory ridges separated into two patches in the male and completely lacking in the female (1), straight and flattened bristles of the tarsus IV comb (11), etc.

Besides, the rugose carapace cuticle (12) and the clearly proximal tarsal organ (13) of *Knoflachia* gen. nov. seem unique among the ‘distal theridiids’.

## Supplementary Material

XML Treatment for Knoflachia

XML Treatment for Knoflachia kurilensis

## ﻿Additional information

### Author ORCIDs

Yuri M. Marusik https://orcid.org/0000-0002-4499-5148

Kirill Y. Eskov https://orcid.org/0000-0002-7953-0746

### Data availability

All of the data that support the findings of this study are available in the main text.
